# Inferring causal genes at type 2 diabetes GWAS loci through chromosome interactions in islet cells

**DOI:** 10.12688/wellcomeopenres.18653.2

**Published:** 2023-08-08

**Authors:** Jason M. Torres, Han Sun, Vibe Nylander, Damien J. Downes, Martijn van de Bunt, Mark I. McCarthy, Jim R. Hughes, Anna L. Gloyn

**Affiliations:** 1 Wellcome Centre for Human Genetics, University of Oxford, Oxford, England, OX3 7BN, UK; 2 Department of Pediatrics, Division of Endocrinology and Diabetes, Stanford University School of Medicine, Stanford, CA, 94305, USA; 3 Oxford Centre for Diabetes Endocrinology & Metabolism, University of Oxford, Oxford, England, OX3 7L3, UK; 4 Medical Research Council Molecular Haematology Unit, Medical Research Council Weatherall Institute of Molecular Medicine, Radcliffe Department of Medicine, University of Oxford, Oxford, OX3 9D2, UK; 5 Present address: Cytoki Pharma ApS, Tuborg Boulevard 12, Hellerup, DK-2900, Denmark; 6 Present address: OMNI Human Genetics, Genentech, 1 DNA Way, South San Francisco, CA, 94080, USA; 7 MRC WIMM Centre for Computational Biology, MRC Weatherall Institute of Molecular Medicine, Radcliffe Department of Medicine, University of Oxford, Oxford, OX3 9D2, UK

**Keywords:** type 2 diabetes, Capture-C, EndoC-βH1 cells, human islets, genetic regulation, chromatin interactions, GWAS loci

## Abstract

**Background: **Resolving causal genes for type 2 diabetes at loci implicated by genome-wide association studies (GWAS) requires integrating functional genomic data from relevant cell types. Chromatin features in endocrine cells of the pancreatic islet are particularly informative and recent studies leveraging chromosome conformation capture (3C) with Hi-C based methods have elucidated regulatory mechanisms in human islets. However, these genome-wide approaches are less sensitive and afford lower resolution than methods that target specific loci.

**Methods: **To gauge the extent to which targeted 3C further resolves chromatin-mediated regulatory mechanisms at GWAS loci, we generated interaction profiles at 23 loci using next-generation (NG) capture-C in a human beta cell model (EndoC-βH1) and contrasted these maps with Hi-C maps in EndoC-βH1 cells and human islets and a promoter capture Hi-C map in human islets.

**Results: **We found improvements in assay sensitivity of up to 33-fold and resolved ~3.6X more chromatin interactions. At a subset of 18 loci with 25 co-localised GWAS and eQTL signals, NG Capture-C interactions implicated effector transcripts at five additional genetic signals relative to promoter capture Hi-C through physical contact with gene promoters.

**Conclusions: **High resolution chromatin interaction profiles at selectively targeted loci can complement genome- and promoter-wide maps.

## Introduction

Most variants implicated by genome-wide association studies (GWAS) are non-coding and are thought to influence type 2 diabetes risk through regulatory effects on gene expression within physiologically relevant cell types. Such regulatory effects may involve allelic consequences at enhancers – DNA elements that increase gene transcription with activity that can vary with cell type, developmental stage, or physiological state. As such, the process of elucidating causal genes (and their corresponding effector transcripts) requires integration of functional genomic and molecular epigenomic information in disease relevant cell types and under relevant conditions. Importantly, regulatory effects on gene expression are facilitated by chromatin interactions and causal genes may be identified by their physical contact with enhancer elements encompassing diabetes-associated variants.

Recent studies have employed methods based on chromatin conformation capture (3C) and implicated genes at GWAS loci associated with type 2 diabetes by mapping chromatin structure in human islets and beta cells ( Greenwald *et al.*, 2019; Lawlor *et al.*, 2019; Miguel-Escalada *et al.*, 2019; Su *et al.*, 2022). All 3C protocols involve the same core key steps – fixation of chromatin with formaldehyde, restriction enzyme digestion, and re-ligation of restriction fragments ( Davies *et al.*, 2017). The resulting “ligation junctions”, which comprise fragments that are co-localised spatially but may be separated linearly by tens to hundreds of kilobases, are sequenced and incorporated into maps of interacting chromatin. Variations in preparing the 3C library and extracting ligation junctions of interest can influence the resolution, sensitivity, and genomic coverage of the resulting chromatin maps, therefore the choice of 3C-based method can markedly alter the detail of chromatin structure information. These differences may affect the inferences made about islet cell biology and the role of T2D associated GWAS variants.

To date, 3C maps in human islets and beta cells have been based on Hi-C approaches that provide genome-wide coverage (and can be enriched for ligation junctions involving promoters), but afford limited detail at individual loci due to prohibitive sequencing requirements and use of low-resolution restriction enzymes ( Davies *et al.*, 2017). Alternatively, the NG capture-C method enables improved resolution and sensitivity at target loci, also referred to as viewpoints or baits, through enrichment from high-resolution 3C libraries ( Davies *et al.*, 2017; Hughes *et al.*, 2014). 

To assess the extent to which chromatin maps generated from different 3C-based methods impact mechanistic inference at T2D GWAS loci, we performed a systematic evaluation of 27 gene promoters at 23 loci. We performed next generation (NG) capture-C, which involves a double capture procedure that can enrich for captured fragments by up to 1,000,000-fold ( Davies *et al.*, 2016), and targeted promoters in the EndoC-βH1 human beta cell line ( Ravassard *et al.*, 2011). We also mapped chromatin interactions using sequenced ligation junctions from recent studies that applied Hi-C in EndoC-βH1 cells and human islets, and promoter-capture (pc) Hi-C in human islets ( Lawlor *et al.*, 2019; Miguel-Escalada *et al.*, 2019). By comparing these maps with those from NG capture-C and incorporating GWAS variants co-localised with expression quantitative trait loci (eQTLs) in human islets, we show how distinct chromatin profiles influence the resolution of causal genes for T2D and glycaemic traits.

## Methods

### Experimental model and external data

EndoC-βH1 cells (RRID: CVCL_L909) were purchased from Endocell, and cultured as described previously ( Hastoy *et al.*, 2018; Ravassard *et al.*, 2011). Lymphoblastoid cell lines (GM12878; RRID: CVCL_7526) were procured from the Coriell Institute. Publicly available *Mbo*I enzyme-based Hi-C sequencing data corresponding to EndoC-βH1 cells and human islets (n=1), were accessed ( Lawlor *et al.*, 2019). Promoter capture Hi-C (pcHi-C) data corresponding to human islets from four donors were downloaded from the EGA database (accession number EGAS00001002917).

### Next generation capture-C

Promoters for 27 gene transcripts at 23 loci were selected for capture. These included 21 genes at 18 loci harbouring both islet eQTLs and genome-wide significant associations with type 2 diabetes and/or glycemic traits ( van de Bunt *et al.*, 2015; Viñuela *et al.*, 2020). The eQTLs referred to for promoter selection included all study-wide significant eQTLs from a mapping study in 118 human islets that were in linkage disequilibrium (1000 Genomes Project CEU *r* ^2^ > 0.8) with GWAS variants ( van de Bunt *et al.*, 2015) (Table S1, *Extended data*). Additional eQTLs from the Integrated Network for Systemic analysis of Pancreatic Islet RNA Expression (InsPIRE) consortium (including eQTLs for *ADCY5*, *TCF7L2*, and *GPSM1*) were also represented in this set ( Viñuela *et al.*, 2020) (Table S1, *Extended data*). The six remaining genes included three control genes with high expression in lymphoblastoid cell lines (LCLs), the *GCK* gene encoding glucokinase (implicated in monogenic forms of diabetes and hyperinsulinemia), and two genes ( *CDKAL1* and *SOX4*) at the *CDKAL1* locus associated with T2D (Table S1, *Extended data*). 70-mer biotinylated oligonucleotide probes (IDT xGen Lockdown oligonucleotides) targeting *Dpn*II restriction fragments were designed using CapSequm ( Hughes *et al.*, 2014) with filtering for repetitive elements, duplicates (≤2), BLAT density score (≤40), and GC content (≤%60) ( Downes *et al.*, 2022).

*In situ* 3C libraries were generated in EndoC-βH1 cells and LCLs by *Dpn*II digestion and T7 ligation chromatin ( Davies *et al.*, 2016). 3C material was sonicated to 200 base pairs (bp) and indexed using NEB Next DNA library prep reagents. Indexed libraries were pooled and double capture was performed with Nimblegen SeqCap EZ reagents (Roche) ( Davies *et al.*, 2016). Sequencing was performed on the Illumina NextSeq platform with 150 bp paired-end reads. Sequenced reads were mapped to GRCh38 with bowtie using CCseqBasicS ( Telenius *et al.*, 2020) which trims adaptor sequences, reconstructs read pairs with flash, conducts an *in silico* digestion of *Dpn*II fragments, maps reads, identifies paired “capture” and “reporter” fragments, and filters PCR duplicates.

### Hi-C and promoter capture Hi-C

Hi-C sequencing data for EndoC-βH1 cells and human islets were processed using the Juicer (v1.75) pipeline ( Durand *et al.*, 2016). Sequencing reads from the *Hind*III-digested pcHi-C library were processed and mapped to genome build GRCh38 using HiCUP (v0.8.1) ( Wingett *et al.*, 2015). Bait and prey fragments were quantified using chicagoTools from the CHiCAGO package (v1.14.0) ( Cairns *et al.*, 2016). Promoter bait design coordinates for genome build hg19 were obtained from a previous study ( Javierre *et al.*, 2016) and lifted over to GRCh38 using LiftOver.

### ATAC-seq

ATAC library preparation and sequencing was performed for nine passages of the EndoC-βH1 cell line using the same protocols used in Thurner *et al.*, 2018 ( Thurner *et al.*, 2018). Human islet ATAC sequencing data corresponding to 13 donors from the Miguel-Escalada *et al.*, 2019 study was accessed from the EGA database (accession number EGAS00001002917). Sequencing reads were processed and mapped to GRCh38 using the ENCODE ATAC-seq bioinformatic pipeline (v1.9.3), with peak calling performed with MACS2 (v2.7.1) ( Feng *et al.*, 2012) using default parameters. Reads within peaks were quantified with featureCounts (v2.0.1) ( Liao *et al.*, 2019) and normalized by median of observed count ratio size factors with DESeq2 (v1.26.0) ( Love *et al.*, 2014).

### Quantification and statistical analysis

NG capture-C reporter counts for each replicate (n=3) of EndoC-βH1 and LCL cells were normalized to the number of *cis* reporter counts ( *i.e.* same chromosome) per 100,000 *cis* reporter reads with CaptureCompare ( Telenius *et al.*, 2020). Chromatin interaction mapping in NG capture-C, pcHi-C, and Hi-C datasets was performed with peaky ( Eijsbouts *et al.*, 2019) using recommended settings (omega = -3.8). Interactions were considered significant if the marginal posterior probability of contact (MPPC) exceeding 0.01 within a range of 250 kb to the viewpoint ( *i.e.* captured fragment) or 0.1 between 250 kb and 1 Mb relative to the viewpoint. Differential chromatin interactions (NG capture-C) between EndoC-βH1 and LCL cells, and differentially accessible (ATAC-seq) peaks between EndoC-βH1 and primary human islet cells were called using DESEq2 (v1.26.0).

## Results

We compared chromatin interaction maps for 27 promotors at 23 loci in human EndoC-βH1 cells, derived from NG capture-C, with previously published Hi-C maps ( Lawlor *et al.*, 2019) in EndoC-βH1 cells and human islets, and with a pcHi-C map ( Miguel-Escalada *et al.*, 2019) in human islets. These experiments showed marked differences in sensitivity with the NG capture-C EndoC-βH1 experiment yielding at least ~27X more ligation junctions than the Hi-C based studies (median = 17,533 ligation junctions; Table S2, *Extended data*). We assessed how these experimental differences impacted our ability to resolve chromatin interactions. We applied a Bayesian model implemented in peaky ( Eijsbouts *et al.*, 2019) to detect fragments showing significant physical interaction with each of the viewpoint ( *a.k.a.* “bait”) fragments encompassing the targeted promoters. peaky extends upon the negative binomial regression model (implemented in methods such as CHiCAGO) and estimates marginal posterior probabilities of contact (MPPC) that indicate most likely chromatin contact sites. Although peaky was developed for Hi-C and pcHi-C data, it can also be applied to 4C and capture-C datasets, and therefore generate interaction probabilities that enable a comparison across 3C-based experiments. However, due to the sparsity of per-fragment ligation junction reads, the peaky algorithm failed to converge (and hence unable to perform statistical tests) for six viewpoints in the pcHi-C islet dataset and for all 27 viewpoints in the Hi-C datasets (Table S3, *Extended data*). In contrast, peaky successfully mapped interactions at all viewpoints in the NG capture-C EndoC-βH1 experiment. After merging adjacent fragments with significant interactions, there were 3.6X as many interactions identified by peaky for NG capture-C than for pcHi-C. Moreover, the median width of significantly interacting chromatin regions was 14.3-fold shorter, indicating a greater ability to fine-map interactions in tandem with increased sensitivity (Table S3, *Extended data*). Notably, this enhanced resolution of significantly interacting regions was influenced by both the use of a 4-bp cutting RE ( *DpnII*) in the NG capture-C experiment rather than the 6-bp cutter *HindIII* used in the pcHi-C experiment, and the greater per-locus sequencing depth afforded by the NG capture-C study design.

**Figure 1.**
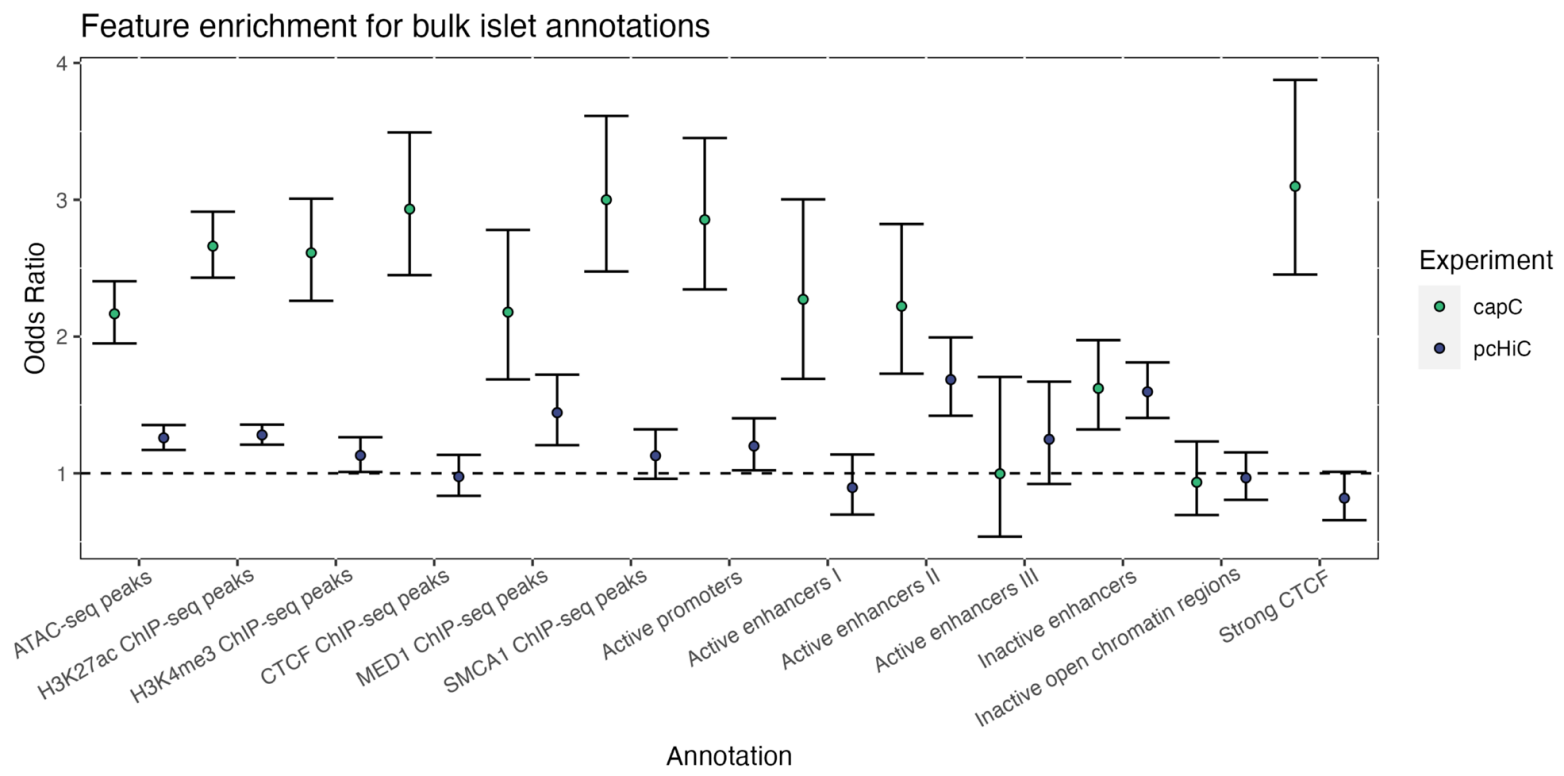
Enrichment of islet epigenomic features at regions with chromatin interactions. Chromatin interactions with the captured promoters from the EndoC-βH1 capture-C (green) and islet pcHi-C (blue) experiments were mapped with peaky and evaluated for enrichment of islet ATAC-seq peaks, histone ChIP-seq peaks, and regulome features from Miguel Escalada *et al*., 2019. Enrichment across all captures was assessed by binning 1kb segments within 1Mb of each targeted promoter and performing Fisher’s exact test for each set of islet features and chromatin interactions. The three negative control viewpoints ( *CR2*, *PAX5*, and *TNFSF11*) were excluded from enrichment analysis.

We next evaluated enrichment of islet regulatory features among interaction peaks resolved by the NG capture-C and pcHi-C experiments and observed significant enrichment for both sets of interactions ( Figure 1). However, given the greater sensitivity and higher resolution of the NG capture-C experiment (which included the use of DpnII), corresponding enrichment estimates were higher for this study. Among this set of interactions, enriched islet features included accessible chromatin peaks (Fisher’s exact test odds ratio [OR]=2.17, 95% CI [1.95, 2.40]), H3K27ac ChIP-seq peaks (OR=2.66, 95% CI [2.43, 2.91]) and active promoter (OR=2.85, 95% CI [2.34, 3.45]) and enhancer elements ( e.g. type 1 active enhancer, OR=2.27, 95% CI [1.69, 3.00]) ( Figure 1). With respect to strong islet enhancer elements (i.e. “Active enhancer I” elements from Miguel-Escalada *et al*., 2019), we found that 9% (34 / 374) of enhancers at the evaluated loci overlap peaky interactions in the NG capture-C experiment, and ~13% (47 / 374) overlap interactions in the pcHi-C experiment (Table S4). This is notable as the median peaky interaction length was 12.15 Kb in the pcHi-C experiment but only 851 bp in the NG capture-C experiment (Table S3). Moreover, ~4% (14 / 370) of additional interactions in the NG capture-C experiment (with respect to interactions in the pcHi-C experiment) overlapped strong enhancer elements, whereas the converse number was ~5% (9 / 165) for pcHi-C interactions. Similarly, we observed that 7% (298 / 4,228) of islet ATAC-seq peaks overlap peaky interactions in the NG capture-C experiment, and ~17% (712 / 4,222) overlap interactions in the pcHi-C experiment (Table S5). Furthermore, ~32% (118 / 370) of additional interactions in the NG capture-C experiment overlapped ATAC-seq peaks, whereas the converse number was ~67% (118 / 165) for pcHi-C interactions. Therefore, an appreciable proportion of additional interactions resolved from the NG capture-C experiment co-localized with relevant islet regulatory features despite have a median width that was over 14X smaller than that for pcHi-C interactions.

**Figure 2.**
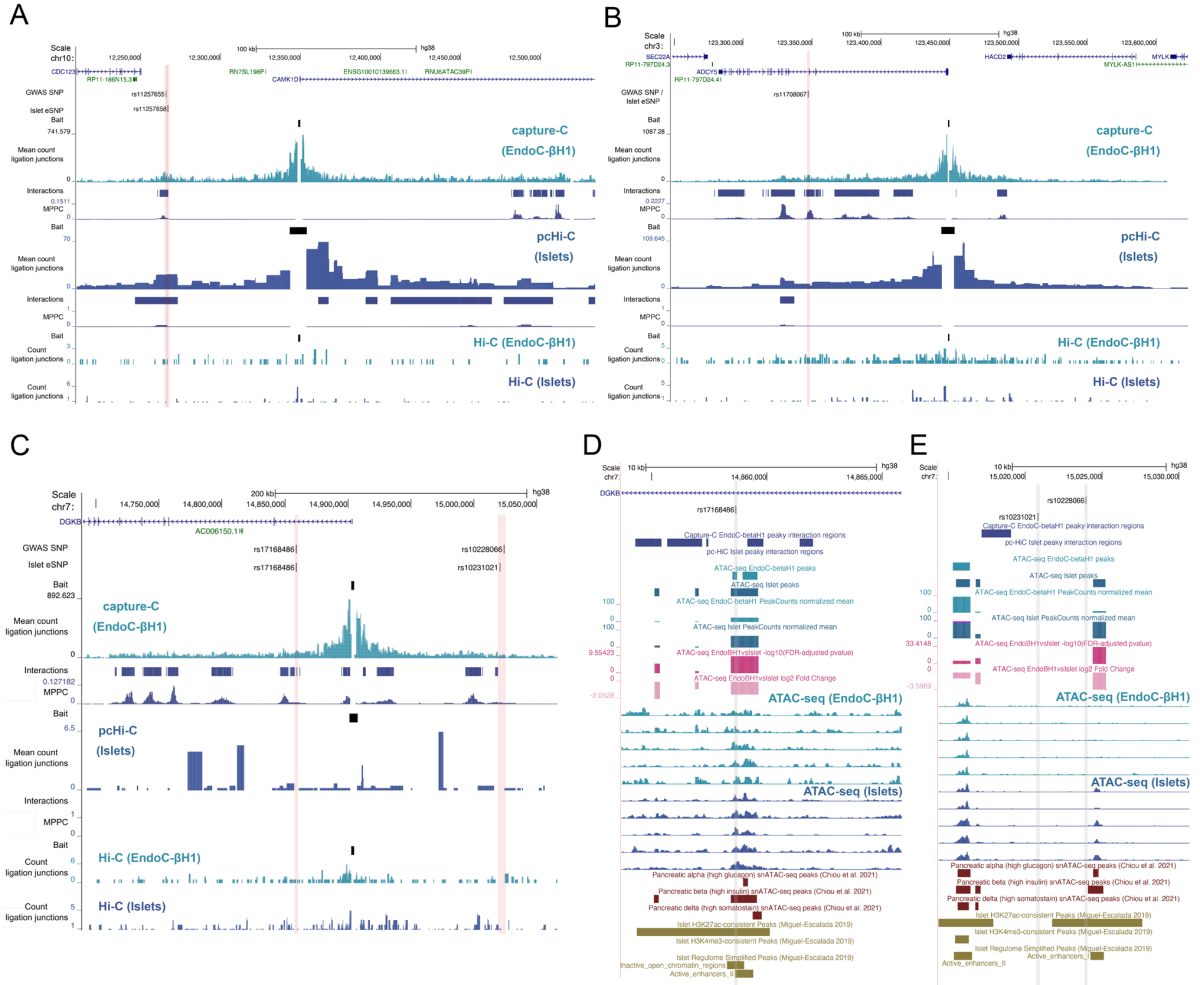
Chromatin interaction profiles at trait-associated loci. Co-localised GWAS-eQTL SNPs are shown with ligation junctions obtained from 3C-based experiments at the ( **A**) *CDC123/CAMK1D*, ( **B**) *ADCY5*, and ( **C**) *DGKB* locus. Tracks of significant chromatin interactions and marginal posterior probability of contact (MPPC) values are shown below the EndoC-βH1 capture-C and islet pcHi-C tracks. Red vertical bars indicate SNP coordinates across 3C-based tracks. Gene annotations correspond to GENCODE V38 protein (blue) and RNA (green) encoding genes. Molecular epigenome profile at the *DGKB* locus is shown for SNPs ( **D**) rs17168486 and ( **E**) rs10231021 and rs10228066. Differential accessibility between EndoC-βH1 and human islets was assessed using DESeq2 and FDR-adjusted -log10 p-values and log2 fold changes are shown in the dark and light pink, respectively. Select single nuclear ATAC-seq peaks in islet alpha, beta, and delta cells from Chiou *et al.*, 2021 are shown in dark red. Histone post-translational modification ChIP-seq peaks and regulome annotations in human islets from Miguel-Escalada *et al.*, 2019 are shown in dark gold. Grey vertical grey bars indicate SNP coordinates across tracks.

To assess how different chromatin interaction profiles impact mechanistic inference at GWAS loci, we integrated single nucleotide polymorphisms (SNPs) associated with islet gene expression ( *i.e.* eSNPs) and type 2 diabetes and/or glycaemic traits. Of the 27 captured promoters, 21 corresponded to eGenes implicated at 18 loci by 25 pairs of co-localised eSNP and GWAS variants (Table S1, *Extended data*). A total of 12 of these co-localised signals were supported by either NG capture-C (n=10) or pcHi-C (n=7), with five receiving support from both methods (Table S6, *Extended data*). Included in this set of five was a signal at the *CAMK1D* locus where a genetic association with type 2 diabetes involving SNP rs11257655 is co-localised with an eQTL involving rs11257658 (linkage disequilibrium *r ^2^* =0.994). The G allele of rs11257658 is associated with decreased human islet expression of *CAMK1D* which encodes calcium/calmodulin-dependent protein kinase 1D ( van de Bunt *et al.*, 2015; Viñuela *et al.*, 2020). Both variants, located ~82 kb upstream of the *CAMK1D* promoter, map to chromatin that physically interacts with the promoter site, thereby corroborating the eQTL ( Figure 2A). Although the resolution in the pc-HiC study was markedly lower than that for capture-C, the interaction maps from both experiments support *CAMK1D* as an effector gene at this locus.

Furthermore, there were five genetic signals supported by chromatin interactions only in our NG capture-C experiment. This included a co-localised eQTL-GWAS signal fine-mapped to a single SNP (rs11708067) at the *ADCY5* locus where the A allele associates with lower islet expression of *ADCY5*, greater T2D risk, and higher levels of fasting glucose ( Dupuis *et al.*, 2010; Morris *et al.*, 2012; van de Bunt *et al.*, 2015; Viñuela *et al.*, 2020; Voight *et al.*, 2010) ( Figure 2B). We previously reported a chromatin interaction at this locus and allelic imbalance where the risk A allele at rs11708067 associated with decreased chromatin accessibility ( Thurner *et al.*, 2018). Another notable example occurred at the *DGKB* locus where there are two independent T2D-associated signals: rs10228066 and rs17168486. While no significant chromatin interactions were detectable at this locus in the pcHi-C and Hi-C experiments due to low signal, multiple interaction peaks were resolved from the NG capture-C data, including peaks near the T2D-associated SNPs. The rs17168486 variant, located ~45 kb upstream of the *DGKB* promoter, mapped within 500 bp of chromatin that significantly interacts with the *DGKB* promoter region ( Figure 2C). Notably, this SNP – where the T2D risk allele T associates with increased expression of *DGKB* in human islets ( Viñuela *et al.*, 2020) – also overlaps enhancer elements in islets and EndoC-βH1 cells (but not in liver, adipose, or skeletal muscle) and accessible chromatin in primary alpha and beta cells ( *i.e.* single-nucleus ATAC-seq peaks) ( Figure 2D). Moreover, this chromatin accessible region was recently predicted to regulate the expression of *DGKB* in human beta cells with high-insulin content ( Chiou *et al.*, 2021), which is further supported by *in vitro* data demonstrating the T2D risk haplotype at rs17168486 influenced luciferase expression in 832/13 and MIN6 cells ( Viñuela *et al.*, 2020). In contrast, the rs10228066 variant, located ~121 kb downstream of the *DGKB* promoter and co-localised with the rs10231021 eQTL signal (LD *r ^2^* =0.881), mapped more than 1.7 kb from the nearest chromatin interaction. Moreover, neither rs10231021 nor rs10228066 directly overlapped accessible chromatin in EndoC-βH1 or islet cell types ( Figure 2E). Furthermore, in a recent trans-ethnic GWAS meta-analysis involving 180,834 T2D cases and 1.159M controls, the rs17168486 signal was fine-mapped to a single variant (rs17168486) whereas the other conditionally independent signal at the *DGKB* locus (where the lead SNP rs2215383 is in strong linkage disequilibrium with rs10228066; *r ^2^* =0.999 in the TOPMED European dataset) was less resolved (13 credible variants and credible interval of 2,318 bp) ( Mahajan *et al.*, 2020) and had a credible interval that did not overlap or map within 500 bp of a chromatin interaction. However, a T2D risk haplotype involving variants in high LD with the rs10231021 eSNP did show higher luciferase expression in 832/13 and MIN6 cells with three variants also showing allele-specific binding in a mobility shift assay ( Viñuela *et al.*, 2020).

**Figure 3.**
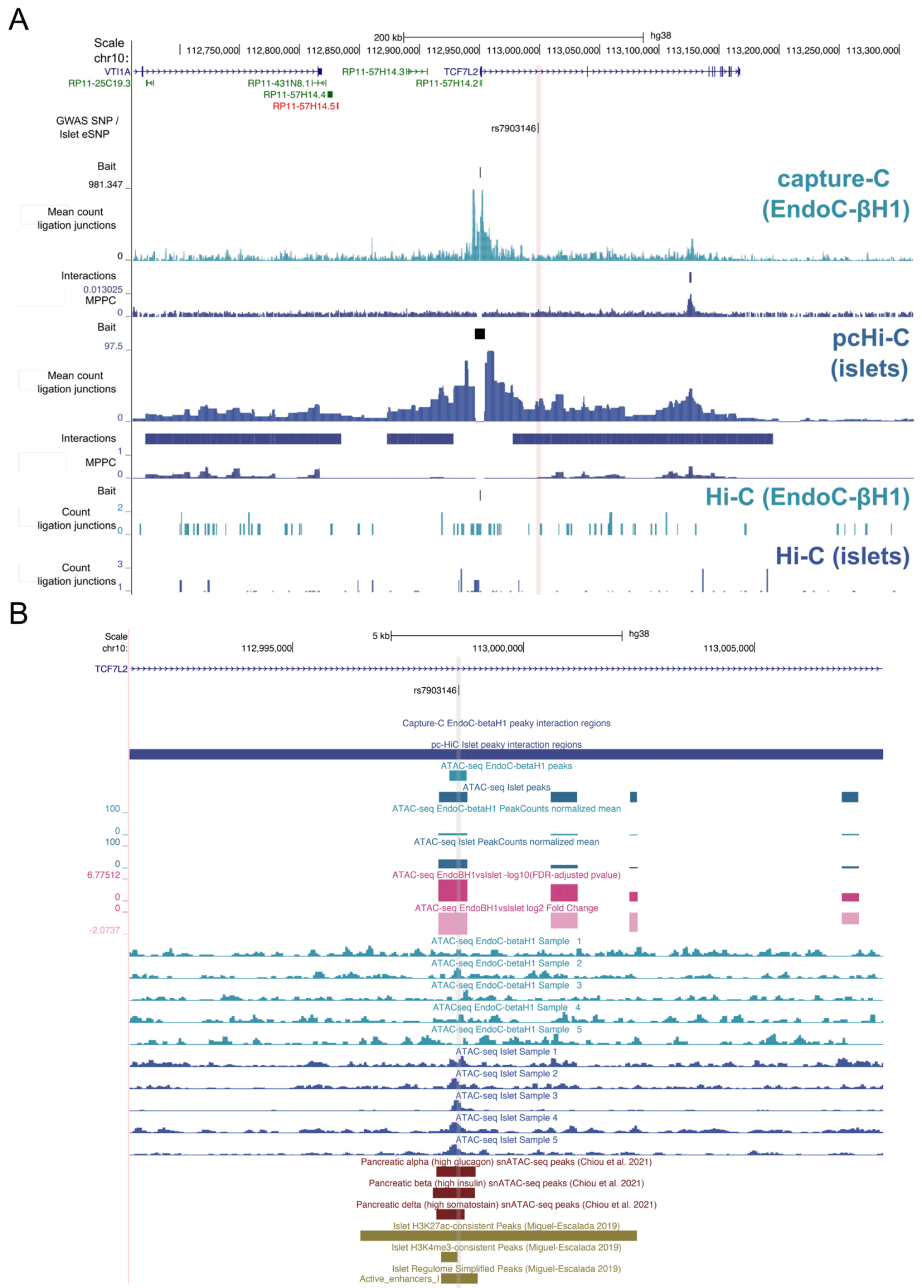
Molecular epigenome profile at the *TCF7L2* locus. ( **A**) Co-localised GWAS-eQTL SNPs are shown with ligation junctions obtained from 3C-based experiments. Tracks of significant chromatin interactions and marginal posterior probability of contact (MPPC) values are shown below the EndoC-βH1 capture-C and islet pcHi-C tracks. Red vertical bars indicate SNP coordinates across 3C-based tracks. Gene annotations correspond to GENCODE V38 protein (blue) and RNA (green) encoding genes. Maps of chromatin accessibility in EndoC-βH1 and human islets are shown for ( **B**) rs7903146. Differential accessibility between EndoC-βH1 and human islets was assessed using DESeq2 and FDR-adjusted -log10 p-values and log2 fold changes are shown in dark and light pink, respectively. Select single nuclear ATAC-seq peaks in islet alpha, beta, and delta cells from Chiou *et al.*, 2021 are shown in dark red. Histone post-translational modification ChIP-seq peaks and regulome annotations in human islets from Miguel-Escalada *et al.*, 2019 are shown in dark gold. Grey vertical bars indicate SNP coordinates across tracks.

Despite the higher resolution afforded by the NG capture-C procedure, there were two genetic signals supported by chromatin interactions only in the pcHi-C experiment in human islets: *TCF7L2* and *UBE2E2* (Table S6, *Extended data*). At the *TCF7L2* locus, which was fine mapped to a single SNP, rs7903146, the T2D risk allele (T) associates with increased *TCF7L2* expression in islets ( Viñuela *et al.*, 2020) and the SNP overlaps an islet enhancer element and accessible chromatin in bulk islet tissue and islet alpha, beta, and delta cells ( Figure 3, Table S7, *Extended data*). Notably, chromatin accessibility at this region was considerably lower in EndoC-βH1 cells than in islets (log2FC=-2.07; FDR-adjusted p-value = 1.68e-07). Therefore, the lower accessibility in this cell-type may explain, in part, the lack of pronounced chromatin interaction at this site from the NG capture-C profile. In the case of *UBE2E2*, a T2D-associated SNP rs35352848 is co-localised with an eQTL (rs13094957) for *UBE2E2* expression in islets and overlapped a broad islet pcHi-C chromatin interaction with the *UBE2E2* promoter. However, neither variant directly maps to H3K27ac peaks, enhancer elements, or accessible chromatin in islets, or in snATAC-seq peaks in beta, alpha, or delta cells (Table S7, *Extended data*). Notably, a recent trans-ancestry GWAS meta-analysis fine-mapped this signal to six credible variants and a wide credible interval of nearly 200 kb (overlapping 12 chromatin interactions). Therefore, more investigation is needed to resolve the causal variant at this signal. 

## Discussion

We found that NG capture-C achieved substantially greater resolution and sensitivity over Hi-C based approaches at the loci we investigated in human beta cells. This corresponded to more refined chromatin interactions that implicated effector transcripts through physical contact between enhancer elements harbouring GWAS variants and gene promoters. Reciprocally, these narrower interaction sites may enhance genetic fine-mapping of variants in contact with a causal gene as they will encompass fewer molecular epigenomic features (e.g. assessible chromatin, H3K27ac ChIP-seq peaks, etc.) than wider interactions gleaned from Hi-C based methods, as can be seen at the *CAMK1D* locus ( Figure 1A and Figure S1, *Extended data*).

 Chromatin interactions provided support for genes implicated by GWAS and eQTL co-localization at 12 of 25 evaluated signals, with five signals supported by interactions in both the pcHi-C and NG capture-C experiments. Relative to the pcHi-C study, interactions from our NG capture-C study corroborated five additional target genes: *ADCY5*, *DGKB*, and three genes at the *GPSM1* locus ( *DNLZ*, *CARD9*, and *GPSM1*). Experimental studies have implicated both *ADCY5* and *DGKB* in insulin secretion, with *ADCY5* shown to be indispensable for glucose-induced insulin secretion in human islets ( Hodson *et al.*, 2014; Peiris *et al.*, 2018). Furthermore, our results corroborate co-localized GWAS and islet eQTL signals at the *GPSM1* locus. Notably, of the signals evaluated in this study, only chromatin encompassing the SNP rs28505901 at this locus showed a significantly stronger interaction in EndoC-βH1 cells relative to LCLs, specifically with the *DNLZ* promoter (Table S6, *Extended data*).

Although the candidate gene promoters targeted in this study were implicated by co-localization between trait-associated GWAS variants and eQTLs in human islets, they do not necessarily represent a comprehensive list of causal genes at these loci. Indeed, pcHi-C interaction maps in human islets, and enhancer perturbation via genome editing in EndoC-βH3 cells, also implicate *OPTN* as an additional effector gene at the *CDC123-CAMK1D* locus (in this case, *OPTN* is a distal gene located more than 830Kb from the trait-associated variant rs11257655) ( Miguel-Escalada *et al*., 2019). However, restricting the analysis to a common set of promoters for genes supported by eQTL co-localization did allow for a direct comparison of interaction profiles between the 3C-based experiments at these loci. Further comparisons based on an expanded set of effector genes supported by additional and disparate lines of evidence (e.g. *in vitro* genomic screens, mouse knock-out studies, etc.) will characterise the difference in interaction profiles gleaned from NG capture-C and pcHi-C to a greater extent. 

Chromatin interactions from the EndoC-βH1 NG capture-C experiment did not corroborate candidate disease genes at all evaluated loci, with *TCF7L2* being the most salient exception. We observed lower chromatin accessibility in EndoC-βH1 cells at this site which may reflect an epigenomic profile corresponding to an earlier developmental stage. Alternatively, EndoC-βH1 cells are a cell line where experimental replicates correspond to a uniform genotype whereas biological replicates from human donors can harbour allelic variation. Notably, the rs7903146 variant was previously found to show evidence of allelic imbalance in a FAIRE-seq experiment in human islet cells where the T (risk-increasing) allele corresponded to a more open chromatin state ( Gaulton *et al*., 2010). On the other, EndoC-βH1 cells are homozygous for the C allele at rs7903146, which associates with a more closed chromatin state in primary islet cells. Therefore, the observed discordance in chromatin interaction profiles at the *TCF7L2* locus may be the consequence of the lack of genetic variation present in EndoC-βH1 cells.

The unavailability of pcHi-C data in EndoC-βH1 cells and NG capture-C data in islets also limited our comparisons as we could not control for cell-type in our evaluation of these two approaches. It is possible that interactions detected in islets but not in EndoC-βH1 cells may reflect enhancer-promoter loops specific to other islets cell types. Notably, a recent study of Hi-C maps in FACS sorted islet cells implicated alpha cells at a T2D-associated signal at the *WFS1* locus, and acinar cells at a signal mapping to the *CPA4* locus ( Su *et al.*, 2022). Differences in interaction profiles between islets and EndoC-βH1 cells may also reflect distinct epigenomic features resulting from SV40LT transduction. Additional chromatin maps will be needed to fully address these questions and recent improvements in NG capture-C technology may make application in rarer cell populations more tractable ( Downes *et al.*, 2021). However, we have demonstrated that markedly enriching 3C libraries for promoters of interest can reveal additional interactions at type 2 diabetes and glycaemic trait-associated loci. Therefore, selective capture of fine-mapped genetic loci may greatly complement genome- or promoter-wide chromatin maps. Moreover, alternative experimental study designs that enrich 3C-libraries for enhancer elements encompassing trait-associated variants, may provide orthogonal information that further resolves effector transcripts at loci implicated by GWAS.

## Data Availability

NG capture-C sequencing data from EndoC-βH1 cells and LCL cells have been deposited on the European Genome-phenome Archive (EGA), which is hosted by the European Bioinformatics Institute of the European Molecular Biology Laboratory (EMBL-EBI) and the Centre for Genomic Regulation (CRG), under accession number EGAS00001006105. ATAC-seq data from EndoC-βH1 have also been deposited on EGA under accession number EGAS00001006105. Further information about EGA can be found on https://ega-archive.org. This paper analyzes existing, publicly available data. Source data and publicly available resources used for this study supporting all findings are detailed in the resources table (see *Extended data*). Open Science Framework (OSF):Extended data for study 'Inferring causal genes at type 2 diabetes GWAS loci through chromosome interactions in islet cells'. Supplemental information supporting this submission can be found on the OSF project page, https://doi.org/10.17605/OSF.IO/KNC6A. This project contains the following extended data: supplemental-tables.xlsx (Tables S1–S7) supplemental figure (Figure S1) resources-table.pdf (Table of data and software resources used in this study) Data are available under the terms of the Creative Commons Attribution 4.0 International license (CC-BY 4.0). All original code for analyses of chromatin interactions has been deposited at Zenodo and is publicly available as of the date of publication. Zenodo: capture-C_promoter-study-0.1.0.zip. https://doi.org/10.5281/zenodo.7043375 ( Torres, 2022)
