# Supplementary material for: Inferring causal genes at type 2 diabetes GWAS loci through chromosome interactions in islet cells
Source: Wellcome Open Res. 2023 Aug 8;8:165. Originally published 2023 Apr 13. [Version 2] doi: 10.12688/wellcomeopenres.18653.2 (PMC10509606; doi:10.12688/wellcomeopenres.18653.2)
Supplement: Peer review history

# Reviewer report: Reviewer response for version 2

**DOI:** 10.21956/wellcomeopenres.21920.r64864

**Published:** 2023-09-19

**Authors:** Mireia Ramos-Rodríguez

**Affiliations:** 1 University Pompeu Fabra, Barcelona, Spain

The authors have effectively addressed my previous comments. I have no further remarks.

Is the work clearly and accurately presented and does it cite the current literature?

Yes

If applicable, is the statistical analysis and its interpretation appropriate?

Partly

Are all the source data underlying the results available to ensure full reproducibility?

Yes

Is the study design appropriate and is the work technically sound?

Yes

Are the conclusions drawn adequately supported by the results?

Partly

Are sufficient details of methods and analysis provided to allow replication by others?

Partly

Reviewer Expertise:

Regulatory genomics, chromatin, computational genomics

I confirm that I have read this submission and believe that I have an appropriate level of expertise to confirm that it is of an acceptable scientific standard.

# Reviewer report: Reviewer response for version 2

**DOI:** 10.21956/wellcomeopenres.21920.r64863

**Published:** 2023-09-07

**Authors:** Inês Cebola

**Affiliations:** 1 Section of Genetics and Genomics, Department of Metabolism, Digestion and Reproduction, Imperial College London, London, England, UK

Excellence response, thank you for addressing all my points. I have no further comments.

Is the work clearly and accurately presented and does it cite the current literature?

Yes

If applicable, is the statistical analysis and its interpretation appropriate?

Partly

Are all the source data underlying the results available to ensure full reproducibility?

Yes

Is the study design appropriate and is the work technically sound?

Yes

Are the conclusions drawn adequately supported by the results?

Partly

Are sufficient details of methods and analysis provided to allow replication by others?

Yes

Reviewer Expertise:

Epigenomics, regulatory genomics, metabolic disease genetics

I confirm that I have read this submission and believe that I have an appropriate level of expertise to confirm that it is of an acceptable scientific standard.

# Reviewer report: Reviewer response for version 1

**DOI:** 10.21956/wellcomeopenres.20683.r57666

**Published:** 2023-06-02

**Authors:** Mireia Ramos-Rodríguez

**Affiliations:** 1 University Pompeu Fabra, Barcelona, Spain

In this article, the authors provide high resolution interaction profiles at several T2D loci, with the aim of leveraging likely regulatory variants affecting gene expression in beta cells. They use NG Capture-C technology in EndoC-βH1 cells and query 27 gene promoters from 23 different loci (3 of them being negative controls). Additionally, they compare their results with other technologies, mainly promoter-capture HiC data obtained from human islets. Authors show how complementing assays with high resolution profiles at candidate genes can inform on potential variants associated with the regulation of that specific gene. The authors focus on T2D, but this methodology could be also extrapolated to other complex diseases. 

 This study has some limitations, mainly 1) the use of a cell line instead of primary tissue, which is already acknowledged by the authors in the discussion, and 2) the fact that, as they state in the introduction, elucidation of causal genes also includes the study of cells in disease-relevant conditions, and in here they query chromatin interactions in cells that are in a “basal” state. Overall, I consider this work to be valuable to the community, as it exemplifies the use of high resolution chromatin contact profiles to fine-map variants to their target genes and highlights the need of this specific assays to complement the results obtained from other types of experiments which query the whole genome, but have significantly lower resolution.

**Main comments**

1. I am curious as to why the authors decided to perform the analysis using candidate gene promoters as viewpoints and not the genetic signals themselves (leading SNPs or eSNPs overlapping islet/beta cell regulatory elements). The pre-selection of promoters somehow implies that they are the causal genes in the locus, although this may not necessarily be true.
2. As the authors state through the manuscript, the resolution of NG Capture-C is much higher than that of pc-HiC or HiC. However, this is mainly caused by the choice of a restriction enzyme: DpnII, used in NG Capture-C is a 4-cutter and HindIII, used in pc-HiC and HiC, is a 6-cutter. Therefore, by definition, the former will yield smaller fragments and thus have higher resolution. This point should be made clearer in the manuscript.
3. Related to the point above, in Figure 2 the authors compare the enrichment of chromatin features in NG Capture-C and in pc-HiC. The methodology they employ is favouring having stronger enrichment in NG Capture-C interactions, as pc-HiC interactions are much larger than the bin they are using (1 kb) and thus, will likely have lots of bins with 0 chromatin features for a single pc-HiC interaction. Additionally, I don’t think that they key point to make here is that that NG Capture-C is more enriched in chromatin features, but rather that its resolution is high enough that it doesn’t contain many in a single interaction bin, which is key to fine-map regulatory variants in contact with a candidate gene. In this case, it might be more important to show that the number of chromatin features (ATAC, H3K27ac peaks, enhancers, etc.) in a single interaction is much smaller in NG Capture-C than in pc-HiC.
4. Regarding the eSNPs used in the analysis, the authors should include either a citation or a methods section detailing the publication they extracted them from or the steps used to pre-select the candidate SNPs. I understand that they specifically select SNPs that are already associated with islet gene expression, and thus are already causal candidates in beta cells. Nonetheless, it could be that some loci has several candidate SNPs and thus, it might be more powerful to use credible sets instead of leading SNPs. Maybe using this approach they would be able to detect more co-localised signals.

**Other comments:**

- I recommend that the tables and figures be ordered as they appear in the text, as it is a bit confusing to follow the article as is. 

Is the work clearly and accurately presented and does it cite the current literature?

Yes

If applicable, is the statistical analysis and its interpretation appropriate?

Partly

Are all the source data underlying the results available to ensure full reproducibility?

Yes

Is the study design appropriate and is the work technically sound?

Yes

Are the conclusions drawn adequately supported by the results?

Partly

Are sufficient details of methods and analysis provided to allow replication by others?

Partly

Reviewer Expertise:

Regulatory genomics, chromatin, computational genomics

I confirm that I have read this submission and believe that I have an appropriate level of expertise to confirm that it is of an acceptable scientific standard, however I have significant reservations, as outlined above.

# Response

**Published:** 2023-08-02

**Authors:** Jason Torres

**Affiliations:** University of Oxford, Nuffield Department of Population Health, UK

**We greatly appreciate the reviewer taking the time to carefully evaluate our manuscript and offering thoughtful criticism and helpful suggestions. Below are listed the complete set of comments from the reviewer with our responses to each concern.**

1. I am curious as to why the authors decided to perform the analysis using candidate gene promoters as viewpoints and not the genetic signals themselves (leading SNPs or eSNPs overlapping islet/beta cell regulatory elements). The pre-selection of promoters somehow implies that they are the causal genes in the locus, although this may not necessarily be true. 
**We strongly concur with the reviewer that the genes targeting in this study, although supported by eQTL co-localisation in human islets, do not necessarily represent an exhaustive list of causal at these loci. eQTL co-localisation with GWAS signals does provide a direct link between associated variants and a candidate gene that is consistent with a causal relationship, but it is not conclusive of one nor would such a list be comprehensive. However, such a list is more likely to be enriched for causal genes and, importantly, did allow for a direct comparison of the NG capture-C experiment with the pcHi-C experiment, which only targeted promoters explicitly. We now address this point in the Discussion. Moreover, we also agree with the reviewer that an alternative design that explicitly targets enhancer elements encompassing GWAS variants would be of great scientific utility in further resolving effector transcripts. We address this point as well in the discussion (the sentences doing so are also highlighted in our response to comment 5 from the other reviewer).**
2. As the authors state through the manuscript, the resolution of NG Capture-C is much higher than that of pc-HiC or HiC. However, this is mainly caused by the choice of a restriction enzyme: DpnII, used in NG Capture-C is a 4-cutter and HindIII, used in pc-HiC and HiC, is a 6-cutter. Therefore, by definition, the former will yield smaller fragments and thus have higher resolution. This point should be made clearer in the manuscript.
**The reviewer is right that the use of a 4-cutter RE will result in smaller restriction fragments and enable greater resolution. Our intended meaning of resolution pertained to the context of mapping significantly interacting chromatin regions, which is influenced by both the size of RE fragments (i.e. the direct consequence of the RE used) and the amount of signal present at each locus (i.e. the consequence of sequencing depth afforded by the assay). We have now aimed to make this point clearer in the Results section by adding this sentence:**
*“Notably, this enhanced resolution of significantly interacting regions was influenced by both the use of a 4-bp cutting RE (DpnII) in the NG capture-C experiment rather than the 6-bp cutter HindIII used in the pcHi-C experiment, and the greater per-locus sequencing depth afforded by the NG capture-C study design.”*
3. Related to the point above, in Figure 2 the authors compare the enrichment of chromatin features in NG Capture-C and in pc-HiC. The methodology they employ is favouring having stronger enrichment in NG Capture-C interactions, as pc-HiC interactions are much larger than the bin they are using (1 kb) and thus, will likely have lots of bins with 0 chromatin features for a single pc-HiC interaction. Additionally, I don’t think that they key point to make here is that that NG Capture-C is more enriched in chromatin features, but rather that its resolution is high enough that it doesn’t contain many in a single interaction bin, which is key to fine-map regulatory variants in contact with a candidate gene. In this case, it might be more important to show that the number of chromatin features (ATAC, H3K27ac peaks, enhancers, etc.) in a single interaction is much smaller in NG Capture-C than in pc-HiC.
**The reviewer rightly points out that the enrichment analysis described in the study is biased to the smaller interaction regions delineated via the NG capture-C experiment.** **Firstly, we have revised the corresponding paragraph in the Results section to properly contextualize this analysis with respect to the difference in restriction enzymes used in the NG capture-C and pcHi-C experiments (see response to comment 3 from the other reviewer, who also voiced a similar concern with the enrichment analysis).** **Secondly, we strongly agree with the reviewer’s observation that narrower interactions afforded by the NG capture-C experiment may advance genetic fine-mapping of regions in contact with gene promoters as they encompass fewer regulatory annotations that could refine causal variants. We revised the Discussion section by removing the previous sentence regarding annotation enrichment and replacing it with the following text:**
*“This corresponded to more refined chromatin interactions that implicated effector transcripts through physical contact between enhancer elements harbouring GWAS variants and gene promoters. Reciprocally, these narrower interaction sites may enhance genetic fine-mapping of variants in contact with a causal gene as they will encompass fewer molecular epigenomic features (e.g. assessible chromatin, H3K27ac ChIP-seq peaks, etc.) than wider interactions gleaned from Hi-C based methods, as can be seen at the CAMK1D locus (Figure 1A and Figure S1, Extended data).”*
**Moreover, we added a supplemental figure to the Extended data that illustrates the encompassing of fewer annotations within an interaction site (as suggested by the reviewer) at the *CAMK1D* locus.**
**Also, as communicated to the other reviewer, we found an error with two of the  annotation files used in the enrichment analysis (e.g. the BED file used for the “Active enhancer I” annotations also included annotations for the “Active enhancer II” and “Active enhancer III” annotations”). This has now been corrected and the enrichment analysis has been re-run. The correct enrichment values are now indicated in the revised Figure 1. Notably, the observed enrichment values for the capture-C experiment for these two annotations changed slightly from the previous analysis (for “Active enhancer I”, the OR value increased from 2.07 to 2.27) with a more pronounced decrease for the pcHi-C experiment (i.e. 95% CI now overlaps 1 for the “Active enhancer I” experiment for the pcHi-C based interactions). The Results has been revised accordingly, and none of the other annotations were impacted by this issue.**
4. Regarding the eSNPs used in the analysis, the authors should include either a citation or a methods section detailing the publication they extracted them from or the steps used to pre-select the candidate SNPs. I understand that they specifically select SNPs that are already associated with islet gene expression, and thus are already causal candidates in beta cells. Nonetheless, it could be that some loci has several candidate SNPs and thus, it might be more powerful to use credible sets instead of leading SNPs. Maybe using this approach they would be able to detect more co-localised signals.
**We appreciate the reviewer’s concern regarding clarifying the source of eQTLs used as reference for selecting promoters to target in this study. We have expanded the methods section to make clear that the set of eQTLs used as the basis for this experiment included all study-wide significant eQTLs in a mapping study of 118 human islets from van de Bunt *et al*. 2015, that were in high LD with GWAS-implicated variants associated with T2D and/or glycaemic traits. Additional eQTLs were considered from an early release of eQTL mapping results from the INSPIRE consortium. This has also been indicated with reference to Table S1 in the Extended data section that includes a full list of loci, genes, GWAS variants, and eQTL SNPs involved in the study design: **
*Promoters for 27 gene transcripts at 23 loci were selected for capture. These included 21 genes at 18 loci harbouring both islet eQTLs and genome-wide significant associations with type 2 diabetes and/or glycemic traits* *(van de Bunt et al., 2015; Viñuela et al., 2020)* *. The eQTLs referred to for promoter selection included all study-wide significant eQTLs from a mapping study in 118 human islets that were in linkage disequilibrium (1000 Genomes Project CEU r ^2^ > 0.8) with GWAS variants* *(van de Bunt et al., 2015)* *(Table S1, Extended data). Additional eQTLs from the Integrated Network for Systemic analysis of Pancreatic Islet RNA Expression (InsPIRE) consortium (including eQTLs for ADCY5, TCF7L2, and GPSM1) were also represented in this set* *(Viñuela et al., 2020)* *(Table S1, Extended data). The six remaining genes included three control genes with high expression in lymphoblastoid cell lines (LCLs), the GCK gene encoding glucokinase (implicated in monogenic forms of diabetes and hyperinsulinemia), and two genes (CDKAL1 and SOX4) at the CDKAL1 locus associated with T2D (Table S1, Extended data).*
**We agree with the reviewer that leveraging information on credible sets would further resolve sets of candidate genes at trait-associated loci to target for chromatin capture. However, at the time the study was initially conceived, genetic fine-mapping had not yet been performed for islet eQTLs, nor was Bayesian co-localisation analysis. Hence, we were restricted to the best available index variants at these loci for considering eGenes (i.e. genes implicated by eQTL associations) to include. We strongly concur that basing future capture studies on fine-mapped credible sets would be highly informative for implicating effector transcripts at GWAS loci. Targeting enhancer elements that intersect with Bayesian credible intervals would be particularly informative for implicating genes, as this would obviate the need to target select promoters at GWAS loci. Although outside the immediate scope of this investigation, the comparison of 3C-ased assays described in this work supports the utility of capture studies in islet cells in complementing promoter-wide surveys of chromatin structure.**
5. Other comments:  I recommend that the tables and figures be ordered as they appear in the text, as it is a bit confusing to follow the article as is.
**Thank you for flagging this issue, which understandably adds confusion. The figure ordering has now been corrected.  **

# Reviewer report: Reviewer response for version 1

**DOI:** 10.21956/wellcomeopenres.20683.r56239

**Published:** 2023-05-23

**Authors:** Inês Cebola

**Affiliations:** 1 Section of Genetics and Genomics, Department of Metabolism, Digestion and Reproduction, Imperial College London, London, England, UK

This study by Torres et al constitutes an interesting addition to the regulatory genomics field, particularly for those interested in disentangling complex GWAS loci. Thorough comparisons such as these are still needed to inform researchers of the best methodologies to employ for specific applications (all vs. a few). The authors studied a total of 18 type 2 diabetes (T2D)-associated loci comparing a targeted capture method (NG Capture-C) versus genome-scale methods, particularly another capture based method called promoter-capture Hi-C. As acknowledged by the authors in the discussion, the study has limitations in terms of number of loci queried and the inherent differences between the cell line used (EndoC bH1) and primary human islets. Nevertheless, using these loci, the authors revealed that NG Capture-C offers superior resolution to investigate specific loci for the majority of cases, opening a new avenue for future molecular investigations of T2D loci.

 In order to improve the manuscript, I have a few suggestions and queries for the authors:

1. Introduction: To the benefit of readers from different fields, I would link better the concepts of “regulatory effects” and “enhancer elements” in the first paragraph, which are provided as somewhat distinct concepts, but it would make sense to make the association between the two more obvious.
2. I recommend reordering the figures, so that they are numbered in order of their first mention in the manuscript.
3. The authors provide a good overview of the key steps of 3C-based protocols in the introduction. In terms of the caveats of these protocols, I would argue that the enzyme used for the digestion step may be the most critical factor to achieve higher or lower resolution in a given experiment. This is actually reflected in the comparisons described in the manuscript: NG Capture-C yielded more and smaller interacting fragments – isn’t this observation expected given the higher frequency of DpnII (used in NG Capture-C) versus HindIII (used in pcHi-C) cut sites in the genome? Similarly, aren’t the results shown in Figure 2 biased towards detecting enrichment for the methods based on smaller interacting fragments (the authors used bins of 1kb, which is smaller than the average fragment size for HindIII). I recommend making these points clearer in the results and discussion sections, as this is a key point to interpret the results presented.
4. The authors employed a method called “peaky” to call significant interactions, observing a superior performance of peaky over for instance CHiCAGO scores. It would be interesting if the authors framed this observation in light of the likelihood that the additional interactions detected by NG Capture-C/peaky are true positives. This can be done by expanding of the description of previous studies comparing the methods, but I would argue that the authors have evidence for this in their dataset as well, given the stronger enrichment of active CRE signatures at interacting regions. It would be of value to the readers to also know more of the actual numbers at play here. For instance, how many new islet enhancer regions are detected as interacting by NG Capture-C, but not by the other methods?
5. When describing the results for the *CAMK1D* locus, the authors refer to it as the “the” effector gene. Although a large bulk of evidence points in that direction, it must be noted that CRISPR epigenome editing experiments of this locus raised the hypothesis that another distal gene ( *OPTN*) could also be affected by disruption of that T2D islet enhancer (Miguel-Escalada et al. 2019). Thus, toning down this statement would be more adequate, as the authors only baited a selection of genes in the locus. This limitation of the study design could be pointed out in the discussion. It would also be interesting to discuss alternative experimental designs for future NG Capture-C experiments, for instance baiting directly enhancers containing SNPs prioritised a priori.
6. For the *TCF7L2* locus comparisons, the authors use it to exemplify some potential limitations of the NG Capture-C albeit, as pointed out, given the small number of tested loci (compared to all the loci associated with T2D) it is difficult to extrapolate for how many loci pcHiC may have a superior performance. The authors could use their sequencing data (e.g. ATAC-seq reads) to check the genotype of the EndoC bH1 cells in relation to the queried SNPs as this may explain some of the discrepancies in the results. For instance, for the specific case of TCF7L2, these cells may simply be homozygous for the allele that associates with reduced regulatory activity. As shown by Gaulton et al (Nat Gen 2010) ^1^, this variant has a marked effect on chromatin accessibility. As EndoC’s are a cell line and thus their experimental replicates represent a single genotype/2 alleles, their allelic distribution may be markedly different from that of a series of islet samples from different donors. Thus, it may not be adequate do carry out a differential accessibility analysis with DESeq2 as shown in Figure 3. It likely makes more sense to frame the level of accessibility in light of the genotype of the cells/samples.

Is the work clearly and accurately presented and does it cite the current literature?

Yes

If applicable, is the statistical analysis and its interpretation appropriate?

Partly

Are all the source data underlying the results available to ensure full reproducibility?

Yes

Is the study design appropriate and is the work technically sound?

Yes

Are the conclusions drawn adequately supported by the results?

Partly

Are sufficient details of methods and analysis provided to allow replication by others?

Yes

Reviewer Expertise:

Epigenomics, regulatory genomics, metabolic disease genetics

I confirm that I have read this submission and believe that I have an appropriate level of expertise to confirm that it is of an acceptable scientific standard, however I have significant reservations, as outlined above.

## References

[1] : A map of open chromatin in human pancreatic islets. *Nat Genet* .2010;42(3) : 10.1038/ng.530 255-9 10.1038/ng.530 20118932PMC2828505

# Response

**Published:** 2023-08-02

**Authors:** Jason Torres

**Affiliations:** University of Oxford, Nuffield Department of Population Health, UK

**We greatly appreciate both reviewers taking the time to carefully evaluate our manuscript and offering thoughtful criticism and helpful suggestions. Below are listed the complete set of comments from each reviewer with our responses to each concern.**

**Reviewer 1:**

 This study by Torres et al constitutes an interesting addition to the regulatory genomics field, particularly for those interested in disentangling complex GWAS loci. Thorough comparisons such as these are still needed to inform researchers of the best methodologies to employ for specific applications (all vs. a few). The authors studied a total of 18 type 2 diabetes (T2D)-associated loci comparing a targeted capture method (NG Capture-C) versus genome-scale methods, particularly another capture based method called promoter-capture Hi-C. As acknowledged by the authors in the discussion, the study has limitations in terms of number of loci queried and the inherent differences between the cell line used (EndoC bH1) and primary human islets. Nevertheless, using these loci, the authors revealed that NG Capture-C offers superior resolution to investigate specific loci for the majority of cases, opening a new avenue for future molecular investigations of T2D loci.  

 In order to improve the manuscript, I have a few suggestions and queries for the authors:

 1.            Introduction: To the benefit of readers from different fields, I would link better the concepts of “regulatory effects” and “enhancer elements” in the first paragraph, which are provided as somewhat distinct concepts, but it would make sense to make the association between the two more obvious.

**We agree that the connection between these related concepts should be made more explicit. The first paragraph has been revised to include the sentence:** ***“*** *Such regulatory effects may involve allelic consequences at enhancers – DNA elements that increase gene transcription with activity that can vary with cell type, developmental stage, or physiological state.” *    

 2.           I recommend reordering the figures, so that they are numbered in order of their first mention in the manuscript.****

**We are grateful to the reviewer for flagging this issue. The figure ordering has now been corrected.  **  

 3.         The authors provide a good overview of the key steps of 3C-based protocols in the introduction. In terms of the caveats of these protocols, I would argue that the enzyme used for the digestion step may be the most critical factor to achieve higher or lower resolution in a given experiment. This is actually reflected in the comparisons described in the manuscript: NG Capture-C yielded more and smaller interacting fragments – isn’t this observation expected given the higher frequency of DpnII (used in NG Capture-C) versus HindIII (used in pcHi-C) cut sites in the genome?  

**The reviewer is correct in that the restriction enzyme (RE) used is the most important factor when it comes to resolution of interaction maps, as this directly determines fragment size. However, our understanding with respect to detecting significantly interacting chromatin regions via formal statistical analysis is that the resolution of such regions will be the consequence of both fragment size (i.e. the RE used in the assay) and the amount of signal present (i.e. the per-locus sequencing depth). We have aimed to make this point clearer by including this sentence in the Results section: **  

*"Notably, this enhanced resolution of significantly interacting regions was influenced by both the use of a 4-bp cutting RE (DpnII) in the NG capture-C experiment rather than the 6-bp cutter HindIII used in the pcHi-C experiment, and the greater per-locus sequencing depth afforded by the NG capture-C study design.”* 

 Similarly, aren’t the results shown in Figure 2 biased towards detecting enrichment for the methods based on smaller interacting fragments (the authors used bins of 1kb, which is smaller than the average fragment size for HindIII). I recommend making these points clearer in the results and discussion sections, as this is a key point to interpret the results presented.

**This is a fair criticism. Given the same binning scheme, the observed enrichment of a fixed set of regulatory annotations among chromatin interactions resolved from different assays will be biased towards those gleaned from smaller RE fragments (i.e. an assay using a RE with a more frequent cutting sequence), provided that both assays are sensitive enough to detect interactions at these regulatory regions. Greater sensitivity to detect interactions at regulatory regions (as we observed for the NG capture-C experiment) will increase enrichment estimates, but such estimates will also be upwardly biased due to the use of *DpnII* rather than *HindIII*.**

**We have now revised the relevant paragraph of the Results section to present this observation in proper context:**

***“*** *We next evaluated enrichment of islet regulatory features among interaction peaks resolved by the NG capture-C and pcHi-C experiments and observed significant enrichment for both sets of interactions (Figure 1). However, given the greater sensitivity and higher resolution of the NG capture-C experiment (which included the use of DpnII), corresponding enrichment estimates were higher for this study. Among this set of interactions, enriched islet features included accessible chromatin peaks (Fisher’s exact test odds ratio [OR]=2.17, 95% CI [1.95, 2.40]), H3K27ac ChIP-seq peaks (OR=2.66, 95% CI [2.43, 2.91]) and active promoter (OR=2.85, 95% CI [2.34, 3.45]) and enhancer elements (e.g. type 1 active enhancer, OR=2.07, 95% CI [1.72, 2.46])(Figure 1).”*

**We have also removed mention of annotation enrichment in the Discussion section and instead emphasize the value of more refined interaction sites in fine-mapping interactions between promoter and elements (and the trait-associated GWAS signals therein), in keeping with our response to comment 3 from the other reviewer.**

**Also, please note that upon reviewing the enrichment analysis in preparing a response to this comment, we found an error with the annotation files generated for the “Active enhancer I” and “Active enhancer II” elements, wherein the set of intervals for these features contained additional intervals that corresponded to other annotations (e.g. the BED file used for the “Active enhancer I” annotations also included annotations for the “Active enhancer II” and “Active enhancer III” annotations”). This has now been corrected  and the enrichment analysis has been re-run. The correct enrichment values are now indicated in the revised Figure 1. Notably, the observed enrichment values for the capture-C experiment for these two annotations changed slightly from the previous analysis (for “Active enhancer I”, the OR value increased from 2.07 to 2.27) with a more pronounced decrease for the pcHi-C experiment (i.e. 95% CI now overlaps 1 for the “Active enhancer I” experiment for the pcHi-C based interactions). The Results has been revised accordingly, and none of the other annotations were impacted by this issue.**  

 4.         The authors employed a method called “peaky” to call significant interactions, observing a superior performance of peaky over for instance CHiCAGO scores. It would be interesting if the authors framed this observation in light of the likelihood that the additional interactions detected by NG Capture-C/peaky are true positives. This can be done by expanding of the description of previous studies comparing the methods, but I would argue that the authors have evidence for this in their dataset as well, given the stronger enrichment of active CRE signatures at interacting regions. It would be of value to the readers to also know more of the actual numbers at play here. For instance, how many new islet enhancer regions are detected as interacting by NG Capture-C, but not by the other methods?  

**We appreciate the reviewer’s comment regarding peaky in relation to CHiCAGO. It is worth emphasizing that we did not perform a direct comparison of these two methods, or intended to argue that peaky is superior to CHiCAGO. In fact, rather than representing two distinct methods for calling interactions, peaky builds on the negative binomial (NB) regression approach implemented in CHiCAGO to jointly model the distribution of NB residuals in a Bayesian fine-mapping framework. In practice, this allowed us to leverage the marginal posterior probabilities of contact (MPPC) values in peaky to focus our comparisons on the most likely chromatin contact sites within the distribution of NB residuals. Notably, peaky was developed in the context of Hi-C and capture Hi-C data, but can also be applied to 4C and NG capture-C data. We have provided further context in the Results section: **  

*“We applied a Bayesian model implemented in peaky* *(Eijsbouts et al., 2019)* *to detect fragments showing significant physical interaction with each of the viewpoint (a.k.a. “bait”) fragments encompassing the targeted promoters. peaky extends upon the negative binomial regression model (implemented in methods such as CHiCAGO) and estimates marginal posterior probabilities of contact (MPPC) that indicate most likely chromatin contact sites. Although peaky was developed for Hi-C and pcHi-C data, it can also be applied to 4C and capture-C datasets, and therefore generate interaction probabilities that enable a comparison across 3C-based experiments. However, due to the sparsity of per-fragment ligation junction reads, the peaky algorithm failed to converge (and hence unable to perform statistical tests) for six viewpoints in the pcHi-C islet dataset and for all 27 viewpoints in the Hi-C datasets (Table S3, Extended data).”*  

**Moreover, we agree with the reviewer’s suggestion to profile the differences in overlap of accessible chromatin and enhancer elements among peaky interactions gleaned from the NG capture-C and pcHi-C experiments. This is now included as additional supplementary tables (Tables S4 and S5 in the revised supplementary tables) and referenced in the Results section: **  

*With respect to strong islet enhancer elements (i.e. “Active enhancer I” elements from Miguel-Escalada et al. 2019), we found that 9% (34 / 374) of enhancers at the evaluated loci overlap peaky interactions in the NG capture-C experiment, and ~13% (47 / 374) overlap interactions in the pcHi-C experiment (Table S4). This is notable as the median peaky interaction length was 12.15 Kb in the pcHi-C experiment but only 851 bp in the NG capture-C experiment (Table S3). Moreover, ~4% (14 / 370) of additional interactions in the NG capture-C experiment (with respect to interactions in the pcHi-C experiment) overlapped strong enhancer elements, whereas the converse number was ~5% (9 / 165) for pcHi-C interactions.  Similarly, we observed that 7% (298 / 4,228) of islet ATAC-seq peaks overlap peaky interactions in the NG capture-C experiment, and ~17% (712 / 4,222) overlap interactions in the pcHi-C experiment (Table S5). Furthermore, ~32% (118 / 370) of additional interactions in the NG capture-C experiment overlapped ATAC-seq peaks, whereas the converse number was ~67% (118 / 165) for pcHi-C interactions.  Therefore, an appreciable proportion of additional interactions resolved from the NG capture-C experiment co-localized with relevant islet regulatory features despite have a median width that was over 14X smaller than that for pcHi-C interactions. *    

 5.         When describing the results for the *CAMK1D* locus, the authors refer to it as the “the” effector gene. Although a large bulk of evidence points in that direction, it must be noted that CRISPR epigenome editing experiments of this locus raised the hypothesis that another distal gene ( *OPTN*) could also be affected by disruption of that T2D islet enhancer (Miguel-Escalada et al. 2019). Thus, toning down this statement would be more adequate, as the authors only baited a selection of genes in the locus. This limitation of the study design could be pointed out in the discussion. It would also be interesting to discuss alternative experimental designs for future NG Capture-C experiments, for instance baiting directly enhancers containing SNPs prioritised a priori.  

**The reviewer is right to point out that Miguel-Escalada et al. 2019 provides compelling evidence from pcHi-C and CRISPR editing experiments for *OPTN* being an effector gene at the *CDC123-CAMK1D *locus, in addition to *CAMK1D* itself. Moreover, the selection of candidate genes (albeit supported by eQTL-colocalisation) targeted in this study does not represent a comprehensive list of all effector genes at these loci. We also strongly agree with the reviewer that there is great value in an alternative experimental design that directly targets trait-associated SNP encompassing enhancer elements. To address these valid points, we first changed the wording in the Results section to:****

*“…interaction maps from both experiments support CAMK1D as **an** effector gene at this locus.” *  

**We have also expanded Discussion sections to address these limitations: **  

*“Although the candidate gene promoters targeted in this study were implicated by co-localization between trait-associated GWAS variants and eQTLs in human islets, they do not necessarily represent a comprehensive list of causal genes at these loci. Indeed, pcHi-C interaction maps in human islets, and enhancer perturbation via genome editing in EndoC-βH3 cells, also implicate OPTN as an additional effector gene at the CDC123-CAMK1D locus (in this case, OPTN is a distal gene located more than 830Kb from the trait-associated variant rs11257655)* *(Miguel-Escalada et al., 2019)* *. However, restricting the analysis to a common set of promoters for genes supported by eQTL co-localization did allow for a direct comparison of interaction profiles between the 3C-based experiments at these loci. Further comparisons based on an expanded set of effector genes supported by additional and disparate lines of evidence (e.g. in vitro genomic screens, mouse knock-out studies, etc.) will characterise the difference in interaction profiles gleaned from NG capture-C and pcHi-C to a greater extent.” *  

***“*** *Moreover, alternative experimental study designs that enrich 3C-libraries for enhancer elements encompassing trait-associated variants, may provide orthogonal information that further resolves effector transcripts at loci implicated by GWAS.” *  

 6.         For the *TCF7L2* locus comparisons, the authors use it to exemplify some potential limitations of the NG Capture-C albeit, as pointed out, given the small number of tested loci (compared to all the loci associated with T2D) it is difficult to extrapolate for how many loci pcHiC may have a superior performance. The authors could use their sequencing data (e.g. ATAC-seq reads) to check the genotype of the EndoC bH1 cells in relation to the queried SNPs as this may explain some of the discrepancies in the results. For instance, for the specific case of *TCF7L2*, these cells may simply be homozygous for the allele that associates with reduced regulatory activity. As shown by Gaulton et al (Nat Gen 2010) 1, this variant has a marked effect on chromatin accessibility. As EndoC’s are a cell line and thus their experimental replicates represent a single genotype/2 alleles, their allelic distribution may be markedly different from that of a series of islet samples from different donors. Thus, it may not be adequate do carry out a differential accessibility analysis with DESeq2 as shown in Figure 3. It likely makes more sense to frame the level of accessibility in light of the genotype of the cells/samples.  

**This is an excellent point brought up by the reviewer that should have been acknowledged in the interpretation of our results. We have revised the relevant section of the Discussion to address this point, which further underscores a key limitation with comparing molecular epigenomic profiles from cell lines directly with those from biological replicates of primary tissue: **  

 ​​​​​​​ ***“*** *Alternatively, EndoC-βH1 cells are a cell line where experimental replicates correspond to a uniform genotype whereas biological replicates from human donors can harbour allelic variation. Notably, the rs7903146 variant was previously found to show evidence of allelic imbalance in a FAIRE-seq experiment in human islet cells where the T (risk-increasing) allele corresponded to a more open chromatin state* *(Gaulton et al., 2010)* *. On the other, EndoC-βH1 cells are homozygous for the C allele at rs7903146, which associates with a more closed chromatin state in primary islet cells. Therefore, the observed discordance in chromatin interaction profiles at the TCF7L2 locus may be the consequence of the lack of genetic variation present in EndoC-βH1 cells.”*
